# Supplementary material for: COZOID: contact zone identifier for visual analysis of protein-protein interactions
Source: BMC Bioinformatics. 2018 Apr 6;19:125. doi: 10.1186/s12859-018-2113-6 (PMC5889581; doi:10.1186/s12859-018-2113-6)
Supplement: Supplementary file 2 — Software build. Executable binary file of the software tool. (ZIP 118,784 kb) [file 12859_2018_2113_MOESM2_ESM.zip › cozoid/platform/docs/testng-6.5.1-javadoc.zip!/org/testng/TestNG.html]

TestNG (TestNG)


---


|  |  |  |
| --- | --- | --- |
| **Overview** \| **Package** \| **Class** \| **Tree** \| **Deprecated** \| **Index** \| **Help** | |  |
| **PREV CLASS**   **NEXT CLASS** | **FRAMES**    **NO FRAMES**     **All Classes** |  |
| SUMMARY: NESTED \| FIELD \| CONSTR \| METHOD | DETAIL: FIELD \| CONSTR \| METHOD |  |


---


## org.testng Class TestNG

```
java.lang.Object
  org.testng.TestNG
```

---

``` public class TestNG extends java.lang.Object ```

This class is the main entry point for running tests in the TestNG framework.
Users can create their own TestNG object and invoke it in many different
ways:

- On an existing testng.xml- On a synthetic testng.xml, created entirely from Java- By directly setting the test classes

You can also define which groups to include or exclude, assign parameters, etc...

The command line parameters are:

- -d outputdir: specify the output directory
- -testclass class\_name: specifies one or several class names
- -testjar jar\_name: specifies the jar containing the tests
- -sourcedir src1;src2: ; separated list of source directories
  (used only when javadoc annotations are used)
- -target
- -groups
- -testrunfactory
- -listener

Please consult documentation for more details.
FIXME: should support more than simple paths for suite xmls

**Author:**
:   Cedric Beust, Alex Popescu

**See Also:**: `usage()`

---

| **Nested Class Summary** | |
| --- | --- |
| `static class` | `TestNG.ExitCodeListener` |


| **Field Summary** | |
| --- | --- |
| `static java.lang.String` | `DEFAULT_COMMAND_LINE_SUITE_NAME`             The default name for a suite launched from the command line |
| `static java.lang.String` | `DEFAULT_COMMAND_LINE_TEST_NAME`             The default name for a test launched from the command line |
| `static java.lang.String` | `DEFAULT_OUTPUTDIR`             The default name of the result's output directory (keep public, used by Eclipse). |
| `static java.lang.Integer` | `DEFAULT_VERBOSE` |
| `protected static int` | `HAS_FAILURE` |
| `protected static int` | `HAS_FSP` |
| `protected static int` | `HAS_NO_TEST` |
| `protected static int` | `HAS_SKIPPED` |
| `protected  long` | `m_end` |
| `protected  long` | `m_start` |
| `protected  java.util.List<XmlSuite>` | `m_suites` |
| `protected  boolean` | `m_useDefaultListeners` |
| `static java.lang.String` | `SHOW_TESTNG_STACK_FRAMES`             System properties |
| `static java.lang.String` | `TEST_CLASSPATH` |


**Constructor Summary**

`TestNG()`
  
          Default constructor.

`TestNG(boolean useDefaultListeners)`
  
          Used by maven2 to have 0 output of any kind come out
of testng.


| **Method Summary** | |
| --- | --- |
| `void` | `addClassLoader(java.lang.ClassLoader loader)`             Add a class loader to the searchable loaders. |
| `void` | `addExecutionListener(IExecutionListener l)` |
| `void` | `addInvokedMethodListener(IInvokedMethodListener listener)` |
| `void` | `addListener(IInvokedMethodListener listener)` |
| `void` | `addListener(IReporter listener)` |
| `void` | `addListener(ISuiteListener listener)` |
| `void` | `addListener(ITestListener listener)` |
| `void` | `addListener(java.lang.Object listener)` |
| `void` | `addMethodSelector(java.lang.String className, int priority)` |
| `protected  void` | `configure(CommandLineArgs cla)`             Configure the TestNG instance based on the command line parameters. |
| `void` | `configure(java.util.Map cmdLineArgs)`             **Deprecated.** *use new configure(CommandLineArgs) method* |
| `IAnnotationTransformer` | `getAnnotationTransformer()` |
| `java.lang.String` | `getConfigFailurePolicy()`             Returns the configuration failure policy. |
| `protected  org.testng.internal.IConfiguration` | `getConfiguration()` |
| `static TestNG` | `getDefault()`             **Deprecated.** *since 5.1* |
| `java.lang.String` | `getDefaultSuiteName()` |
| `java.lang.String` | `getDefaultTestName()` |
| `protected  long` | `getEnd()` |
| `java.lang.String` | `getOutputDirectory()` |
| `java.util.Set<IReporter>` | `getReporters()` |
| `java.util.List<ITestNGListener>` | `getServiceLoaderListeners()` |
| `protected  long` | `getStart()` |
| `int` | `getStatus()` |
| `java.util.List<ISuiteListener>` | `getSuiteListeners()` |
| `java.lang.Integer` | `getSuiteThreadPoolSize()` |
| `java.util.List<ITestListener>` | `getTestListeners()` |
| `boolean` | `hasFailure()` |
| `boolean` | `hasFailureWithinSuccessPercentage()` |
| `boolean` | `hasSkip()` |
| `void` | `initializeSuitesAndJarFile()` |
| `static boolean` | `isJdk14()`             **Deprecated.** |
| `static void` | `main(java.lang.String[] argv)`             The TestNG entry point for command line execution. |
| `static TestNG` | `privateMain(java.lang.String[] argv, ITestListener listener)`             **Note**: this method is not part of the public API and is meant for internal usage only. |
| `void` | `run()`             Run TestNG. |
| `java.util.List<ISuite>` | `runSuitesLocally()`             This needs to be public for maven2, for now..At least until an alternative mechanism is found. |
| `void` | `setAnnotationTransformer(IAnnotationTransformer t)` |
| `void` | `setCommandLineSuite(XmlSuite suite)` |
| `void` | `setConfigFailurePolicy(java.lang.String failurePolicy)`             Sets the policy for whether or not to ever invoke a configuration method again after it has failed once. |
| `void` | `setDataProviderThreadCount(int count)` |
| `void` | `setDefaultSuiteName(java.lang.String defaultSuiteName)` |
| `void` | `setDefaultTestName(java.lang.String defaultTestName)` |
| `void` | `setExcludedGroups(java.lang.String groups)`             Define which groups will be excluded from this run. |
| `void` | `setGroupByInstances(boolean b)` |
| `void` | `setGroups(java.lang.String groups)`             Define which groups will be included from this run. |
| `void` | `setHasFailure(boolean hasFailure)`             **Deprecated.** *since 5.1* |
| `void` | `setHasFailureWithinSuccessPercentage(boolean hasFailureWithinSuccessPercentage)`             **Deprecated.** *since 5.1* |
| `void` | `setHasSkip(boolean hasSkip)`             **Deprecated.** *since 5.1* |
| `void` | `setJUnit(java.lang.Boolean isJUnit)`             Specify if this run should be made in JUnit mode |
| `void` | `setListenerClasses(java.util.List<java.lang.Class> classes)`             Define which listeners to user for this run. |
| `void` | `setMaster(java.lang.String fileName)`             Specify if this run should be in Master-Slave mode as Master |
| `void` | `setMethodInterceptor(IMethodInterceptor methodInterceptor)` |
| `void` | `setMixed(java.lang.Boolean isMixed)`             Specify if this run should be made in mixed mode |
| `void` | `setObjectFactory(java.lang.Class c)` |
| `void` | `setObjectFactory(ITestObjectFactory factory)` |
| `void` | `setOutputDirectory(java.lang.String outputdir)`             Sets the output directory where the reports will be created. |
| `void` | `setParallel(java.lang.String parallel)`             Define whether this run will be run in parallel mode. |
| `void` | `setPreserveOrder(boolean b)` |
| `void` | `setRandomizeSuites(boolean randomizeSuites)` |
| `void` | `setServiceLoaderClassLoader(java.net.URLClassLoader ucl)` |
| `void` | `setSkipFailedInvocationCounts(java.lang.Boolean skip)` |
| `void` | `setSlave(java.lang.String fileName)`             Specify if this run should be in Master-Slave mode as slave |
| `void` | `setSourcePath(java.lang.String path)`             This method is invoked by Maven's Surefire, only remove it once Surefire has been modified to no longer call it. |
| `void` | `setSuiteThreadPoolSize(java.lang.Integer suiteThreadPoolSize)` |
| `void` | `setTestClasses(java.lang.Class[] classes)`             Set the test classes to be run by this TestNG object. |
| `void` | `setTestJar(java.lang.String jarPath)`             Sets a jar containing a testng.xml file. |
| `void` | `setTestNames(java.util.List<java.lang.String> testNames)`             Only run the specified tests from the suite. |
| `static void` | `setTestNGVersion()`             **Deprecated.** *The TestNG version is now established at load time. This method is not required anymore and is now a no-op.* |
| `protected  void` | `setTestRunnerFactory(ITestRunnerFactory itrf)` |
| `void` | `setTestSuites(java.util.List<java.lang.String> suites)`             Set the suites file names to be run by this TestNG object. |
| `void` | `setThreadCount(int threadCount)`             Define the number of threads in the thread pool. |
| `void` | `setUseDefaultListeners(boolean useDefaultListeners)`             If this method is passed true before run(), the default listeners will not be used. |
| `void` | `setVerbose(int verbose)`             Sets the level of verbosity. |
| `void` | `setXmlPathInJar(java.lang.String xmlPathInJar)`             Sets the path to the XML file in the test jar file. |
| `void` | `setXmlSuites(java.util.List<XmlSuite> suites)`             Specifies the XmlSuite objects to run. |
| `protected static void` | `validateCommandLineParameters(CommandLineArgs args)`             Double check that the command line parameters are valid. |

**Methods inherited from class java.lang.Object**

`clone, equals, finalize, getClass, hashCode, notify, notifyAll, toString, wait, wait, wait`

**Field Detail**

### DEFAULT\_COMMAND\_LINE\_SUITE\_NAME

```
public static final java.lang.String DEFAULT_COMMAND_LINE_SUITE_NAME
```

:   The default name for a suite launched from the command line

    **See Also:**: Constant Field Values

---


### DEFAULT\_COMMAND\_LINE\_TEST\_NAME

```
public static final java.lang.String DEFAULT_COMMAND_LINE_TEST_NAME
```

:   The default name for a test launched from the command line

    **See Also:**: Constant Field Values

---


### DEFAULT\_OUTPUTDIR

```
public static final java.lang.String DEFAULT_OUTPUTDIR
```

:   The default name of the result's output directory (keep public, used by Eclipse).

    **See Also:**: Constant Field Values

---


### SHOW\_TESTNG\_STACK\_FRAMES

```
public static final java.lang.String SHOW_TESTNG_STACK_FRAMES
```

:   System properties

    **See Also:**: Constant Field Values

---


### TEST\_CLASSPATH

```
public static final java.lang.String TEST_CLASSPATH
```

**See Also:**: Constant Field Values

---


### m\_suites

```
protected java.util.List<XmlSuite> m_suites
```

---


### m\_useDefaultListeners

```
protected boolean m_useDefaultListeners
```

---


### HAS\_FAILURE

```
protected static final int HAS_FAILURE
```

**See Also:**: Constant Field Values

---


### HAS\_SKIPPED

```
protected static final int HAS_SKIPPED
```

**See Also:**: Constant Field Values

---


### HAS\_FSP

```
protected static final int HAS_FSP
```

**See Also:**: Constant Field Values

---


### HAS\_NO\_TEST

```
protected static final int HAS_NO_TEST
```

**See Also:**: Constant Field Values

---


### DEFAULT\_VERBOSE

```
public static final java.lang.Integer DEFAULT_VERBOSE
```

---


### m\_end

```
protected long m_end
```

---


### m\_start

```
protected long m_start
```


**Constructor Detail**

### TestNG

```
public TestNG()
```

:   Default constructor. Setting also usage of default listeners/reporters.

---


### TestNG

```
public TestNG(boolean useDefaultListeners)
```

:   Used by maven2 to have 0 output of any kind come out
    of testng.

    **Parameters:**: `useDefaultListeners` - Whether or not any default reports should be added to tests.


**Method Detail**

### getStatus

```
public int getStatus()
```

---


### setOutputDirectory

```
public void setOutputDirectory(java.lang.String outputdir)
```

:   Sets the output directory where the reports will be created.

    :   **Parameters:**: `outputdir` - The directory.

---


### setUseDefaultListeners

```
public void setUseDefaultListeners(boolean useDefaultListeners)
```

:   If this method is passed true before run(), the default listeners
    will not be used.

    - org.testng.reporters.TestHTMLReporter- org.testng.reporters.JUnitXMLReporter- org.testng.reporters.XMLReporter

    :   **See Also:**: `TestHTMLReporter`, `JUnitXMLReporter`, `XMLReporter`

---


### setTestJar

```
public void setTestJar(java.lang.String jarPath)
```

:   Sets a jar containing a testng.xml file.

    :   **Parameters:**: `jarPath` -

---


### setXmlPathInJar

```
public void setXmlPathInJar(java.lang.String xmlPathInJar)
```

:   Sets the path to the XML file in the test jar file.

---


### initializeSuitesAndJarFile

```
public void initializeSuitesAndJarFile()
```

---


### setThreadCount

```
public void setThreadCount(int threadCount)
```

:   Define the number of threads in the thread pool.

---


### setParallel

```
public void setParallel(java.lang.String parallel)
```

:   Define whether this run will be run in parallel mode.

---


### setCommandLineSuite

```
public void setCommandLineSuite(XmlSuite suite)
```

---


### setTestClasses

```
public void setTestClasses(java.lang.Class[] classes)
```

:   Set the test classes to be run by this TestNG object. This method
    will create a dummy suite that will wrap these classes called
    "Command Line Test".

    If used together with threadCount, parallel, groups, excludedGroups than this one must be set first.

    :   **Parameters:**: `classes` - An array of classes that contain TestNG annotations.

---


### addMethodSelector

```
public void addMethodSelector(java.lang.String className,
                              int priority)
```

---


### setTestSuites

```
public void setTestSuites(java.util.List<java.lang.String> suites)
```

:   Set the suites file names to be run by this TestNG object. This method tries to load and
    parse the specified TestNG suite xml files. If a file is missing, it is ignored.

    :   **Parameters:**: `suites` - A list of paths to one more XML files defining the tests. For example: ``` TestNG tng = new TestNG(); List suites = Lists.newArrayList(); suites.add("c:/tests/testng1.xml"); suites.add("c:/tests/testng2.xml"); tng.setTestSuites(suites); tng.run(); ```

---


### setXmlSuites

```
public void setXmlSuites(java.util.List<XmlSuite> suites)
```

:   Specifies the XmlSuite objects to run.

    :   **Parameters:**: `suites` - **See Also:**: `XmlSuite`

---


### setExcludedGroups

```
public void setExcludedGroups(java.lang.String groups)
```

:   Define which groups will be excluded from this run.

    :   **Parameters:**: `groups` - A list of group names separated by a comma.

---


### setGroups

```
public void setGroups(java.lang.String groups)
```

:   Define which groups will be included from this run.

    :   **Parameters:**: `groups` - A list of group names separated by a comma.

---


### setTestRunnerFactory

```
protected void setTestRunnerFactory(ITestRunnerFactory itrf)
```

---


### setObjectFactory

```
public void setObjectFactory(java.lang.Class c)
```

---


### setObjectFactory

```
public void setObjectFactory(ITestObjectFactory factory)
```

---


### setListenerClasses

```
public void setListenerClasses(java.util.List<java.lang.Class> classes)
```

:   Define which listeners to user for this run.

    :   **Parameters:**: `classes` - A list of classes, which must be either ISuiteListener, ITestListener or IReporter

---


### addListener

```
public void addListener(java.lang.Object listener)
```

---


### addListener

```
public void addListener(IInvokedMethodListener listener)
```

---


### addListener

```
public void addListener(ISuiteListener listener)
```

---


### addListener

```
public void addListener(ITestListener listener)
```

---


### addListener

```
public void addListener(IReporter listener)
```

---


### addInvokedMethodListener

```
public void addInvokedMethodListener(IInvokedMethodListener listener)
```

---


### getReporters

```
public java.util.Set<IReporter> getReporters()
```

---


### getTestListeners

```
public java.util.List<ITestListener> getTestListeners()
```

---


### getSuiteListeners

```
public java.util.List<ISuiteListener> getSuiteListeners()
```

---


### setVerbose

```
public void setVerbose(int verbose)
```

:   Sets the level of verbosity. This value will override the value specified
    in the test suites.

    :   **Parameters:**: `verbose` - the verbosity level (0 to 10 where 10 is most detailed) Actually, this is a lie: you can specify -1 and this will put TestNG in debug mode (no longer slicing off stack traces and all).

---


### run

```
public void run()
```

:   Run TestNG.

---


### addExecutionListener

```
public void addExecutionListener(IExecutionListener l)
```

---


### runSuitesLocally

```
public java.util.List<ISuite> runSuitesLocally()
```

:   This needs to be public for maven2, for now..At least
    until an alternative mechanism is found.

---


### getConfiguration

```
protected org.testng.internal.IConfiguration getConfiguration()
```

---


### main

```
public static void main(java.lang.String[] argv)
```

:   The TestNG entry point for command line execution.

    :   **Parameters:**: `argv` - the TestNG command line parameters. **Throws:**: `java.io.FileNotFoundException`

---


### privateMain

```
public static TestNG privateMain(java.lang.String[] argv,
                                 ITestListener listener)
```

:   **Note**: this method is not part of the public API and is meant for internal usage only.

---


### configure

```
protected void configure(CommandLineArgs cla)
```

:   Configure the TestNG instance based on the command line parameters.

---


### setSuiteThreadPoolSize

```
public void setSuiteThreadPoolSize(java.lang.Integer suiteThreadPoolSize)
```

---


### getSuiteThreadPoolSize

```
public java.lang.Integer getSuiteThreadPoolSize()
```

---


### setRandomizeSuites

```
public void setRandomizeSuites(boolean randomizeSuites)
```

---


### setSourcePath

```
public void setSourcePath(java.lang.String path)
```

:   This method is invoked by Maven's Surefire, only remove it once
    Surefire has been modified to no longer call it.

---


### configure

```
@Deprecated
public void configure(java.util.Map cmdLineArgs)
```

:   **Deprecated.** *use new configure(CommandLineArgs) method*

    :   This method is invoked by Maven's Surefire to configure the runner,
        do not remove unless you know for sure that Surefire has been updated
        to use the new configure(CommandLineArgs) method.

---


### setTestNames

```
public void setTestNames(java.util.List<java.lang.String> testNames)
```

:   Only run the specified tests from the suite.

---


### setSkipFailedInvocationCounts

```
public void setSkipFailedInvocationCounts(java.lang.Boolean skip)
```

---


### setMaster

```
public void setMaster(java.lang.String fileName)
```

:   Specify if this run should be in Master-Slave mode as Master

    :   **Parameters:**: `fileName` - remote.properties path

---


### setSlave

```
public void setSlave(java.lang.String fileName)
```

:   Specify if this run should be in Master-Slave mode as slave

    :   **Parameters:**: `fileName` - remote.properties path

---


### setJUnit

```
public void setJUnit(java.lang.Boolean isJUnit)
```

:   Specify if this run should be made in JUnit mode

    :   **Parameters:**: `isJUnit` -

---


### setMixed

```
public void setMixed(java.lang.Boolean isMixed)
```

:   Specify if this run should be made in mixed mode

---


### setTestNGVersion

```
@Deprecated
public static void setTestNGVersion()
```

:   **Deprecated.** *The TestNG version is now established at load time. This
    method is not required anymore and is now a no-op.*

---


### isJdk14

```
@Deprecated
public static boolean isJdk14()
```

:   **Deprecated.**

    :   Returns true if this is the JDK 1.4 JAR version of TestNG, false otherwise.

        :   **Returns:**: true if this is the JDK 1.4 JAR version of TestNG, false otherwise.

---


### validateCommandLineParameters

```
protected static void validateCommandLineParameters(CommandLineArgs args)
```

:   Double check that the command line parameters are valid.

---


### hasFailure

```
public boolean hasFailure()
```

:   **Returns:**: true if at least one test failed.

---


### hasFailureWithinSuccessPercentage

```
public boolean hasFailureWithinSuccessPercentage()
```

:   **Returns:**: true if at least one test failed within success percentage.

---


### hasSkip

```
public boolean hasSkip()
```

:   **Returns:**: true if at least one test was skipped.

---


### getOutputDirectory

```
public java.lang.String getOutputDirectory()
```

---


### getAnnotationTransformer

```
public IAnnotationTransformer getAnnotationTransformer()
```

---


### setAnnotationTransformer

```
public void setAnnotationTransformer(IAnnotationTransformer t)
```

---


### getDefaultSuiteName

```
public java.lang.String getDefaultSuiteName()
```

:   **Returns:**: the defaultSuiteName

---


### setDefaultSuiteName

```
public void setDefaultSuiteName(java.lang.String defaultSuiteName)
```

:   **Parameters:**: `defaultSuiteName` - the defaultSuiteName to set

---


### getDefaultTestName

```
public java.lang.String getDefaultTestName()
```

:   **Returns:**: the defaultTestName

---


### setDefaultTestName

```
public void setDefaultTestName(java.lang.String defaultTestName)
```

:   **Parameters:**: `defaultTestName` - the defaultTestName to set

---


### setConfigFailurePolicy

```
public void setConfigFailurePolicy(java.lang.String failurePolicy)
```

:   Sets the policy for whether or not to ever invoke a configuration method again after
    it has failed once. Possible values are defined in `XmlSuite`. The default
    value is `XmlSuite.SKIP`.

    :   **Parameters:**: `failurePolicy` - the configuration failure policy

---


### getConfigFailurePolicy

```
public java.lang.String getConfigFailurePolicy()
```

:   Returns the configuration failure policy.

    :   **Returns:**: config failure policy

---


### getDefault

```
@Deprecated
public static TestNG getDefault()
```

:   **Deprecated.** *since 5.1*

---


### setHasFailure

```
@Deprecated
public void setHasFailure(boolean hasFailure)
```

:   **Deprecated.** *since 5.1*

---


### setHasFailureWithinSuccessPercentage

```
@Deprecated
public void setHasFailureWithinSuccessPercentage(boolean hasFailureWithinSuccessPercentage)
```

:   **Deprecated.** *since 5.1*

---


### setHasSkip

```
@Deprecated
public void setHasSkip(boolean hasSkip)
```

:   **Deprecated.** *since 5.1*

---


### setMethodInterceptor

```
public void setMethodInterceptor(IMethodInterceptor methodInterceptor)
```

---


### setDataProviderThreadCount

```
public void setDataProviderThreadCount(int count)
```

---


### addClassLoader

```
public void addClassLoader(java.lang.ClassLoader loader)
```

:   Add a class loader to the searchable loaders.

---


### setPreserveOrder

```
public void setPreserveOrder(boolean b)
```

---


### getStart

```
protected long getStart()
```

---


### getEnd

```
protected long getEnd()
```

---


### setGroupByInstances

```
public void setGroupByInstances(boolean b)
```

---


### setServiceLoaderClassLoader

```
public void setServiceLoaderClassLoader(java.net.URLClassLoader ucl)
```

---


### getServiceLoaderListeners

```
public java.util.List<ITestNGListener> getServiceLoaderListeners()
```


---


|  |  |  |
| --- | --- | --- |
| **Overview** \| **Package** \| **Class** \| **Tree** \| **Deprecated** \| **Index** \| **Help** | |  |
| **PREV CLASS**   **NEXT CLASS** | **FRAMES**    **NO FRAMES**     **All Classes** |  |
| SUMMARY: NESTED \| FIELD \| CONSTR \| METHOD | DETAIL: FIELD \| CONSTR \| METHOD |  |


---
